# Relapse after conservative surgery combined with triptorelin acetate versus conservative surgery only in women with focal adenomyosis: study protocol for a multicenter, prospective, randomized controlled trial

**DOI:** 10.1186/s13063-020-04294-2

**Published:** 2020-04-28

**Authors:** Wenwen Wang, Xiangyi Ma, Wei Zhang, Zhiying Li, Yan Wang, Zhiying Yu, Chunlian Zhang, Li Hong, Ruoyu Luo, Hui Xing, Wuliang Wang, Qingfen Yue, Jia Wei, Minli Zhang, Shixuan Wang

**Affiliations:** 1 Department of Obstetrics and Gynecology, Tongji Hospital, Tongji Medical College, Huazhong University of Science and Technology, Wuhan, Hubei China; 2 Department of Obstetrics and Gynecology, Zhongnan Hospital of Wuhan University, Wuhan University, Wuhan, Hubei China; 3 Department of Obstetrics and Gynecology, Affiliated Renhe Hospital, China Three Gorges University, Yichang, Hubei China; 4 Maternal and Child Hospital of Hubei Province, Tongji Medical College, Huazhong University of Science and Technology, Wuhan, Hubei China; 5 Department of Obstetrics and Gynecology, Shenzhen Second People’s Hospital, Shenzhen, China; 6 Department of Obstetrics and Gynecology, Affiliated Taihe Hospital of Hubei University of Medicine, Shiyan, Hubei China; 7 Department of Obstetrics and Gynecology, Renmin Hospital of Wuhan University Hubei General Hospital, Wuhan, Hubei China; 8 Department of Obstetrics and Gynecology, Xiangyang Central Hospital, Hubei University of Arts and Science, Xiangyang, Hubei China; 9 Department of Obstetrics and Gynecology, The Second Affiliated Hospital of Zhengzhou University, Zhengzhou, Henan China; 10 Department of Obstetrics and Gynecology, Luoyang Central Hospital Affiliated to Zhengzhou University, Luoyang, Henan China

**Keywords:** Focal adenomyosis, Adenomyomectomy, Relapse, Triptorelin acetate

## Abstract

**Background:**

The preservation of fertility and integrity of the reproductive organs has increasingly been of concern to most women with adenomyosis. Adenomyomectomy is conservative surgery that is now widely applied; however, relapse is a serious problem after the operation. Postoperative treatment, such as gonadotropin-releasing hormone agonist (GnRHa) has been suggested to result in reducing the rate of disease recurrence. However, there is still a lack of evidence from randomized clinical trials examining the efficacy of GnRHa in decreasing the postoperative recurrence rate.

**Method/design:**

Relapse after conservative surgery combined with triptorelin acetate versus conservative surgery only in women with focal adenomyosis is a multicenter, prospective, randomized controlled trial. The primary outcome is relapse assessed using a visual analogue scale (VRS) and numeric rating scale (NRS), pictorial blood loss assessment chart (PBAC) score, and the size of the uterus and the lesion as measured by two/three-dimensional color doppler ultrasonography (2D/3D-CDUS) or magnetic resonance imaging (MRI). The secondary outcomes include quality of life, clinical pregnancy, ovarian reserve, adverse events, assessment by the Short Form (36) Health Survey and Female Sexual Function index, serum follicle-stimulating hormone, estradiol levels, and anti-Muellerian hormone and so on. All these indexes are measured at 3, 6, 12, 18, 24, 30, and 36 months after conservative surgery.

**Discussion:**

The result of this large, multicenter randomized trial will provide evidence for one of the strategies of long-term management in focal adenomyosis after conservative operation.

**Trial registration:**

Chinese Clinical Trial Registry: ChiCTR1800014340. Registered on 6 January 2018.

## Background

Adenomyosis is a common gynecologic benign disorder characterized by aberrant presence of endometrial glands and stromal cells within the myometrium [1–3]. The main symptoms are dysmenorrhea, chronic pelvic pain, dyspareunia, abnormal uterine bleeding (especially with heavy menstrual bleeding) and infertility, which seriously affect quality of life and work productivity in women of reproductive age [4, 5].

Hysterectomy is considered as radical therapy for patients who are refractory to other treatments or who do not wish to preserve their fertility [6, 7]. However, many patients of reproductive age are very concerned to preserve their fertility and reproductive organs [8]. Medical treatments or conservative surgery are options for these patients. The former include non-steroidal anti-inflammatory drugs or hormone regulating drugs, which can maintain a hypoestrogenic state, such as gonadotropin releasing hormone agonist (GnRHa), danazol, progestogens, or oral contraceptive pills [9, 10]. GnRHa has been the best acceptable treatment for patients during the past 20 years [11]. Unfortunately, these medicines may only provisionally improve symptoms and often lead to serious side effects [12]. Therefore, conservative surgery is an alternative; depending on the extent of the disease, the surgical options are adenomyomectomy (for localized adenomyosis) and partial adenomyomectomy (for diffuse adenomyosis) [4, 13]. The rate of pain relief after conservative surgery is higher in patients with focal adenomyosis than in those with diffuse adenomyosis [14]. However, discrepancy of relapse rate was the main issue for this treatment, due to the residual ratio of lesions. The cause was put down to the unclear cut line between focus and normal myometrial tissue [14]. How to control recurrence after surgery has become the current priority.

Recently, some prospective or retrospective studies [7, 15, 16] have confirmed that surgical-medical treatment is a more effective option for the treatment of symptomatic relief in focal adenomyosis than surgical treatment alone. However, there is still a lack of evidence from randomized controlled studies to confirm whether GnRH agonists could decrease the disease recurrence rate after conservative surgery in women with focal adenomyosis. This study is a multicenter, randomized, parallel controlled trial comparing the efficacy of triptorelin acetate in protecting against postoperative relapse in patients with focal adenomyosis.

## Method/design

Participants (*n* = 308) who have undergone complete excision of focal adenomyosis will be randomly assigned 1:1 to the treatment group (triptorelin acetate) or the control group. Patients will be recruited at 10 hospitals across mid-west China. All research units have been approved by the ethics committees. Every patient will sign an informed consent form prior to this study. This report follows the Standard Protocol Items: Recommendations for Interventional Trials (SPIRIT) guideline (Additional file 1).

### Inclusion criteria

The inclusion criteria are as follows:

1. Women aged ≥ 18 and ≤ 45 years
2. Women with pathologically confirmed diagnosis of focal adenomyosis
3. Women who accept complete excision of adenomyosis and have not taken any steroid hormone therapy in the 3 months before surgery
4. Women who are healthy before and are not pregnant
5. Women who can comply with the study procedures and give written informed consent

### Exclusion criteria

1. Women who are also participating in other clinical trials at the same time
2. Women who have concomitantly been diagnosed with ovarian endometrioma, deep infiltrating endometriosis, or multiple leiomyomas
3. Women with congenital uterine abnormalities such as uterine malformation (unicornis uterus, septate uterus, or duplex uterus) or acute genital inflammation or malignant tumor
4. Women who are pregnant
5. Women who have taken steroid hormone therapy in the 3 months before surgery
6. Women with hereditary disease, blood disease, liver or kidney dysfunction, or malnutrition disorders that cause anemia
7. Women, who may get contraindications, cannot tolerate surgery or are allergic to triptorelin acetate
8. Women who are unable to comply with the study procedures and give written informed consent

### Screening and enrollment

Previous medical history and current medication status are reviewed in the standardized case report forms. A physical examination and imaging such as two/three-dimensional color doppler ultrasonography (2D/3D-CDUS) or magnetic resonance imaging (MRI) are performed. Laboratory measurements include measurement of serum follicle-stimulating hormone (FSH), estradiol (E2), anti-Muellerian hormone (AMH), cancer antigen 125 (CA125), and safety assays including routine analysis of blood, urine, liver function, renal function, hepatitis virus, HIV, syphilis, and coagulation; electrocardiogram (ECG) and radiography are performed in the local departments at the study sites. Either laparoscopy or transabdominal surgery is performed to completely remove all clinically recognizable lesions in patients who are suspected to have focal adenomyosis.

Written informed consent will be obtained from the patients after surgery. Quality of life, degree of pain, and menstrual volume will be recorded using the Short Form (36) Health Survey (SF-36), Female Sexual Function Index (FSFI), pictorial blood loss assessment chart (PBAC) score, visual analogue scale (VAS) and numeric rating scale (NRS) after confirming by histologic assessment. A schedule of enrollment, interventions, and assessment is provided Table 1.

**Table 1:** Schedule of screening, enrollment and assessment

Evaluation	Screening	Enrollment	Time of follow up after surgery (month)						
			3	6	12	18	24	30	36
Written consent		√							
Inclusion/exclusion criteria		√							
Medical history	√								
Physical exam	√								
Safety test (preoperative examination)	√								
2D/3D-CDUS/MRI	√		√	√	√	√	√	√	√
FSH, E_2_, AMH, CA125	√		√	√	√	√	√	√	√
Side effects			√	√	√	√	√	√	√
VAS and NRS		√	√	√	√	√	√	√	√
SF-36 and FSFI		√	√	√	√	√	√	√	√
PBAC score		√	√	√	√	√	√	√	√

*2D/3D-CDUS/MRI* two/three-dimensional color doppler ultrasonography/magnetic resonance imaging, *FSH* follicle stimulating hormone, *E*_*2*_ estradiol, *AMH* anti-Muellerian hormone, *CA125* cancer antigen 125, *VAS* visual analogue scale, *NRS* numeric rating scale, *SF-36* Short Form-36 Health Survey, *FSFI* Female Sexual Function index, *PBAC* pictorial blood loss assessment chart

### Adenomyomectomy

Laparotomy, hysteroscopy, or laparoscopy are performed in this study. During the laparoscopy, patients are in the lithotomy position under general anesthesia with endotracheal intubation. After sterilization, a pneumoperitoneum is created using carbon dioxide gas at 13 mmHg (Karl Storz GmbH & Co. KG, Tuttlingen, Germany). Surgery is performed using four surgical trocars. The supine position and a median incision are suitable for laparotomy. Hysteroscopic surgery is conducted for type 0 or type 1 adenomyoma (Karl Storz GmbH & Co. KG, Tuttlingen, Germany). The surgical approach is adenomyomectomy. The principles of these surgical options include complete removal of clinically visible lesions, maintaining the integrity of the uterine wall, and retaining the integrity of the uterine cavity as far as possible. The duration of surgery, size of the focus, blood loss, and the integrity of the uterine cavity should be recorded.

### Interventions

Eligible participants are assigned by simple randomization in a 1:1 ratio to a group receiving surgery only (group A) or to a group receiving surgery plus GnRH agonists (group B). The GnRH agonist is administered in the intervention group as a 3.75 mg intramuscular injection (Diphereline, Ipsen, France) on the first day of menstruation after surgery, then once every 4 weeks for six courses. The signs and symptoms of hypoestrogenism, including hot flushes, night sweats, sleep disorders, abnormal emotions and osteoporosis, could be caused by the GnRH agonist; these should be carefully evaluated and participants could be treated with tibolone 1.25 mg per day as add-back therapy to maintain serum estradiol at 30–50 pg/ml [17].

The intervention will be discontinued or modified if an intolerable adverse reaction occurs in group B. Color doppler ultrasonography and a steroid hormone test on the 3rd and 6th month after conservative surgery can help to monitor adherence. During the trial, other medications, such as steroid hormone or herbal medicine, or another intervention, such as levonorgestrel-releasing intrauterine system (LNG-IUS) will be prohibited.

### Randomization

Eligible participants are assigned by simple randomization in a 1:1 ratio to one of two groups. The sequence of randomization has been set up by biostatisticians in the data coordination center using Microsoft Excel and the “RAND ()” function. The original sequence is safely kept by the staff in the data coordination center, and it has been put into the online central randomization system by these staff members, who are not involved in enrolling subjects. The sequence is not accessible to any investigators or study coordinators. If a subject fulfills the enrollment criteria, the authorized study coordinator will obtain the assignment for her. After randomization, both subjects and investigators are informed about the assignments.

### Outcome and outcome assessments

The primary outcome is relapse, which is defined as recurrence of dysmenorrhea or pelvic pain, or menorrhagia, or local recurrence compared to the first follow up after surgery as confirmed by 2D/3D-CDUS/MRI. Relapse as identified by 2D/3D-CDUS or MRI constitutes an increase > 1 cm in the maximal diameter of the suspected recrudescent focus during the follow-up period. The VRS and NRS are also applied for evaluation of dysmenorrhea or pelvic pain and the recurrence is defined as any increase in the scores and by participants asking for other medical treatments for relief of symptoms at the same time during follow up. The PBAC score is used to predict coagulation disorders in women with menorrhagia and recurrence is defined as a score > 100. The proportion of participants with recurrence at 3, 6, 9,12, 18, 24, 30, and 36 months will be recorded.

The secondary outcomes include quality of life, clinically confirmed pregnancy, ovarian reserve, and adverse events (AE). Quality of life is assessed by the SF-36 and FSFI, as measures of health status and sexual functioning in women. Ovarian reserve is evaluated by measuring FSH, E_2_, and AMH; the first two are measured before surgery and during the follow-up period at the beginning of the menstrual cycle unless the participant is currently receiving treatment with triptorelin acetate. AMH can be measured at any time before and after surgery at the time of follow up. Differences in scores for eight dimensions (physical functioning, role-physical, bodily pain, general health, vitality, social functioning, role emotional and mental health) of the SF-36 and for the FSFI, and the mean values of FSH, E_2_, and AMH for will be recorded for assessment of quality of life and ovarian reserve participants in each group of participants at 3, 6, 9,12, 18, 24, 30, and 36 months. The participant’s intention to become pregnant, the pregnancy rate, and outcome are recorded in a standard case report form, including details of abortion, premature delivery, and full-term delivery, to evaluate differences in pregnancy outcomes in each treatment arm at 36 months.

The follow-up procedure is acquired from the outpatient department records. Follow-up visits occur at 3, 6, 12, 18, 24, 30, and 36 months after conservative surgery.

Treatment-related AE are monitored at each visit. The AE are any unfavorable medical occurrences associated with the subject’s participation in the research, whether or not considered related to the study intervention. Serious adverse events (SAE) are events that are temporally associated with the subject’s participation in research that meet any of the following criteria: death, events that are life-threatening or severely or permanently disabling, events requiring in-patient hospitalization or prolongation of existing hospitalization, or any events deemed as serious by the local principal investigator. The proportion of patients with AE will be compared at 36 months to determine whether there is a difference between the groups.

### Data analysis

#### Sample size calculation

Sample size was determined based on data from a prospective study demonstrating a relapse rate of about 49% in the group that received surgery alone and 28.1% in the group that received surgery and GnRH agonist treatment [7]. A two-tailed test with alpha set at 0.05 and 85% power is used to detect a minimum clinically meaningful difference between control and intervention groups. The minimal sample size is calculated as 139 participants in each group. In consideration of a dropout rate of 10%, we will recruit 308 participants in total.

#### Data collection

Data are collected using a standard case report form. Data are de-identified before being entered into the database. The study site is regularly monitored and the database checked to ensure the accuracy of data collected. The data management, monitoring, and reporting of this study will comply with the International Conference on Harmonisation Good Clinical Practice (ICH-GCP) guidelines.

#### Data analysis plan

**Fig. 1:**
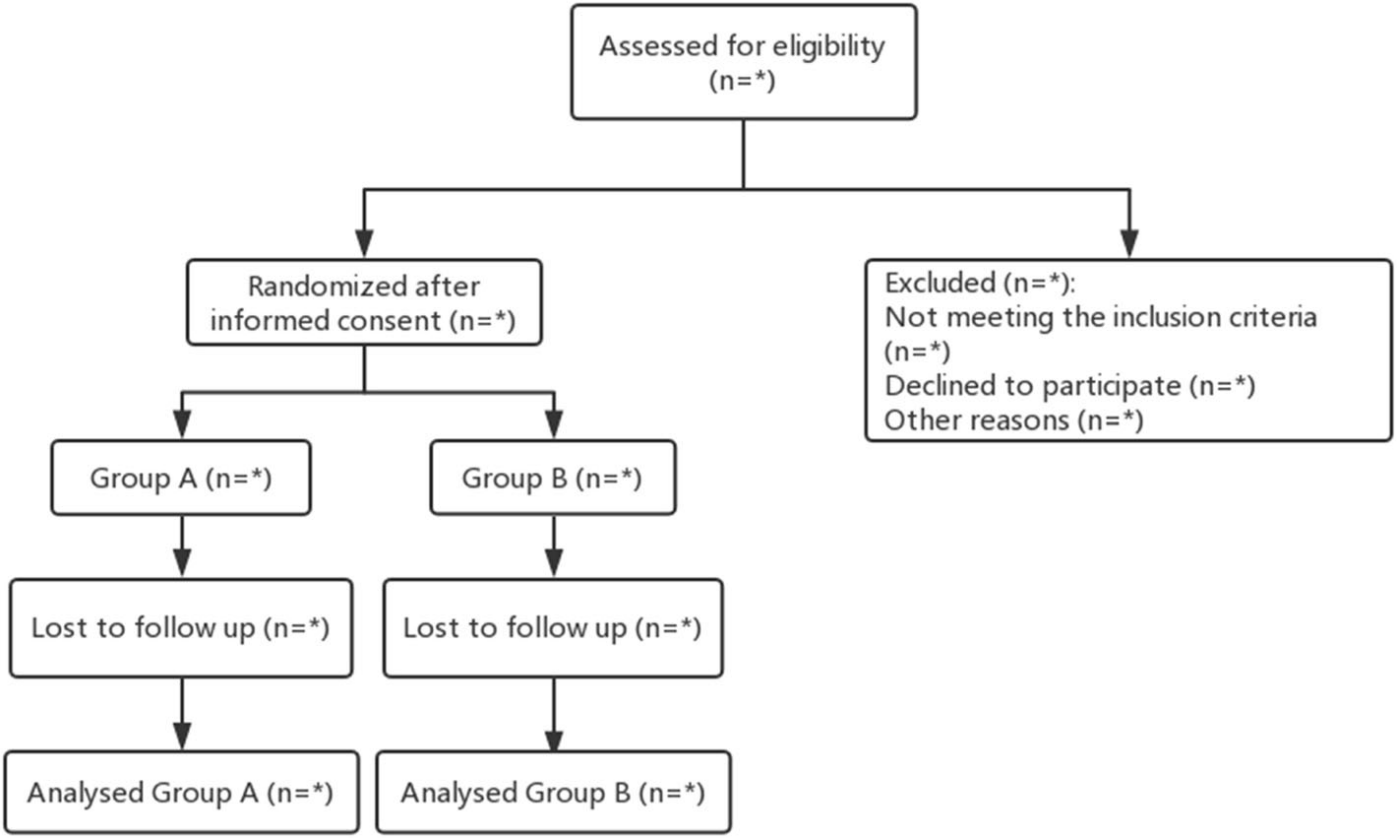
Flowchart of this study

Data analysis and reporting will be conducted in accordance with the Consolidated Standards of Reporting Trials (CONSORT) 2010 Statement, as shown in our flow chart (Fig. 1), including the number of eligible participants and the number lost to follow up for various reasons. Data analysts will be blinded to the participants’ assigned interventions. Unblinding of a participant’s allocated intervention during the trial is permissible after blind verification and a submission of data locking proof by statistical analyst.

Intention to treat, which is based on the initial treatment intent, will be used as a foundation in our analysis. Participants who begin the allocated treatment are part of the trial, whether they finish it or not. The characteristics at baseline will be compared between the control and intervention groups. Continuous data will be summarized by the mean and standard deviation and the Wilcoxon rank sum test will be used to identify differences in baseline characteristic between the two groups. Categorical data will be described by number and percentage, using the Pearson chi-square test to compare data from the two groups.

The primary outcome measure is the difference in the recurrence rate between group A and group B after 3 years of follow up, which will be analyzed by the Pearson chi-square test. For efficacy parameters, such as the score for pelvic pain and dysmenorrhea, menstrual blood loss, and the size of the uterus and the lesion, will be analyzed using generalized estimating equations (GEE) or mixed effects model repeated measures (MMRM) to account for correlation among these observations at different follow-up points.

The variables from secondary outcomes, including scores on the SF-36 and the FSFI, the pregnancy rate, and pregnancy outcomes, are calculated during 36 months of follow up using GEE or MMRM analysis to compare groups A and B to identify differences between groups at different time points. The number of participants with adverse events (AE) or serious adverse events (SAE) will be calculated in each arm and taken into final statistical testing.

#### Dissemination

The results of the study will be published in a peer-reviewed medical journal without using the services of a professional writer. Once agreed by the Steering Committee, the source data will be made available to share through national or international anonymized datasets.

## Discussion

The preservation of fertility and the integrity of the reproductive organs is increasingly of concern to women with adenomyosis. Due to the disadvantage of medical treatments, adenomyomectomy was now increasingly applied. Remission from dysmenorrhea or dyspareunia after complete excision of the lesion has been reported as 50–94.7% and improvement in menorrhagia as 25–80% [18]. Nevertheless, a symptomatic or local recurrence of the condition may occur. About 2.8–13.95% of patients relapsed at the end of first year in this study; 14.28–49% of patients had recurrence of disease in the following 24 months [18]. Therefore, the long-term management after surgery is the main issue to be addressed.

Recently, AI Jama retrospectively analyzed 18 patients who underwent adenomyomectomy and treatment with GnRHa for 24 weeks, 15 of whom had systematic improvement after 1 year of follow up [16]. Liu carried out a non-controlled descriptive study of 186 women with pathologically proven adenomyoma, who underwent ultramini-laparoscopic adenomyomectomy and a 6-month course of goserelin acetate treatment. The rate of systematic recurrence was 9% after 3 years of follow up [19]. Wang et al. identified a significant decline in the relapse rate in the surgical-medical treatment group at the end of 2-year follow up in a prospective, non-randomized study [7]. However, our retrospective analysis showed no difference between the control and intervention group at the end of the first or second year. It is noteworthy that there was a significant difference between the control and the GnRH agonist group in symptomatic relapse or relapse as identified on imaging studies at the end of the 36-months follow up. In other words, postoperative treatment might be applied to reduce the long-term recurrence rate. Whether or not we should use the postoperative method to prevent relapse in patients with adenomyosis is still unknown.

This is the first multicenter, prospective, randomized controlled trial, comparing the efficacy of GnRH agonist in reducing recurrence in patients with focal adenomyosis who have undergone adenomyomectomy. We cannot blind the trial participants and care providers to treatment allocation due to the drug-induced amenorrhea and/or series of side effects after GnRHa therapy, which will help patients and doctors identify the treatment. The lack of blinding might cause substantial bias, especially in the subjective judgment of patients, such pain scoring for the primary outcome. To solve this problem, we set up two types of pain scoring system to reduce the chance of subjective error judgment among patients. In addition, recurrence of dysmenorrhea is defined according to the patient’s pain score and the need for changes in treatment, which can better evaluate progress after conservative treatment. We plan to enroll 308 participants from 10 teaching hospitals in China. The enrollment began in March 2018. At the time of manuscript preparation, more than 90 subjects have been enrolled. The result of this large multicenter randomized trial will provide level-I evidence for the strategy of long-term management of focal adenomyosis after conservative surgery.

### Trial status

The protocol version is Version 1.0; March 8, 2018. Recruitment began on 8 March 2018. The expected date for recruitment completion is October–November 2019.

## Supplementary information

**Additional file 1.** SPIRIT checklist.

## Data Availability

No additional data available.

## Abbreviations

2D/3D-CDUS: Two/three-dimensional color doppler ultrasonography
AMH: Anti-Muellerian hormone
E2: Estradiol
FSFI: Female Sexual Function index
FSH: follicle-stimulating hormone
GEE: Generalized estimating equations
GnRH: Gonadotropin-releasing hormone
MMRM: Mixed effects model repeated measures
MRI: Magnetic resonance imaging
NRS: Numeric rating scale
PBAC: Pictorial blood loss assessment chart
SF-36: Short Form (36) Health Survey
VAS: Visual analogue scale
